# The Role of PTX3 in Mineralization Processes and Aging-Related Bone Diseases

**DOI:** 10.3389/fimmu.2020.622772

**Published:** 2021-01-29

**Authors:** Umberto Tarantino, Chiara Greggi, Ida Cariati, Virginia Veronica Visconti, Monica Gasparini, Marco Cateni, Elena Gasbarra, Annalisa Botta, Antonietta Salustri, Manuel Scimeca

**Affiliations:** ^1^ Department of Clinical Science and Translational Medicine, University of Rome “Tor Vergata”, Rome, Italy; ^2^ Department of Orthopedics and Traumatology, Policlinico Tor Vergata (PTV) Foundation, Rome, Italy; ^3^ Department of Biomedicine and Prevention, University of Rome “Tor Vergata”, Rome, Italy; ^4^ PhD students’ Program in Medical-Surgical and Biotechnologies and Translational Medicine, Faculty of Medicine and Surgery, University of Rome “Tor Vergata”, Rome, Italy

**Keywords:** PTX3, inflammation, mineralization, bone diseases, ectopic calcification, fracture healing, osteoporosis, aging

## Abstract

The Long Pentraxin 3 (PTX3) is a multifunctional glycoprotein released by peripheral blood leukocytes and myeloid dendritic cells in response to primary pro-inflammatory stimuli, that acts as a non-redundant component of the humoral arm of innate immunity. In addition to the primary role in the acute inflammatory response, PTX3 seems to be involved in other physiological and pathological processes. Indeed, PTX3 seems to play a pivotal role in the deposition and remodeling of bone matrix during the mineralization process, promoting osteoblasts differentiation and activity. Recently, PTX3 was seen to be involved in the ectopic calcifications’ formation in breast cancer disease. In this regard, it has been observed that breast cancer tumors characterized by high expression of PTX3 and high amount of Breast Osteoblast Like Cells (BOLCs) showed several Hydroxyapatite (HA) microcalcifications, suggesting a likely role for PTX3 in differentiation and osteoblastic activity in both bone and extra-bone sites. Furthermore, given its involvement in bone metabolism, several studies agree with the definition of PTX3 as a molecule significantly involved in the pathogenesis of age-related bone diseases, such as osteoporosis, both in mice and humans. Recent results suggest that genetic and epigenetic mechanisms acting on *PTX3* gene are also involved in the progression of these diseases. Based on these evidences, the aim of our systemic review was to offer an overview of the variety of biological processes in which PTX3 is involved, focusing on bone mineralization, both in a physiological and pathological context.

## Introduction

**Figure 1:**
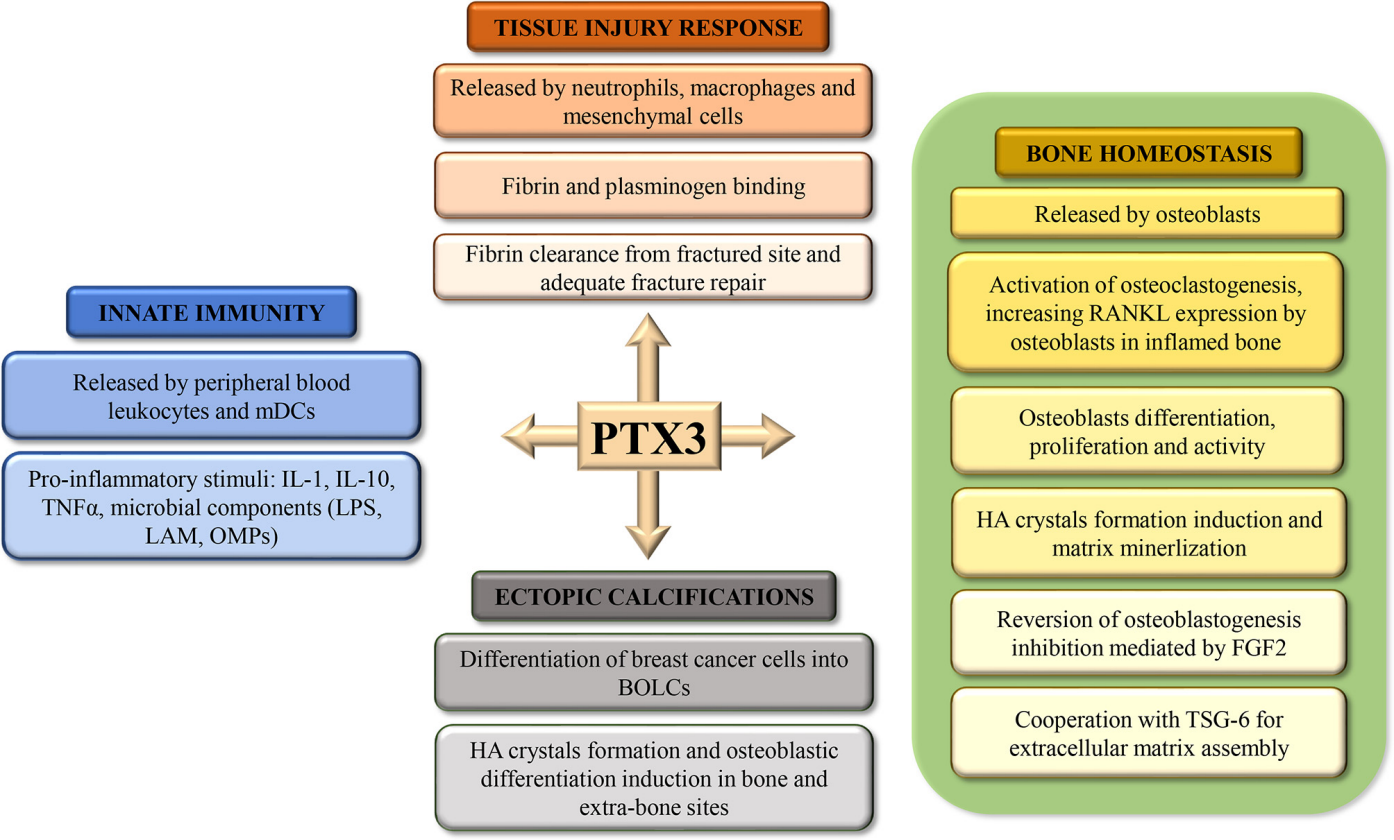
Schematic representation of the roles of Pentraxin 3 (PTX3) in tissue injury response, bone homeostasis, ectopic calcification, and innate immunity.

The Long Pentraxin 3 (PTX3) is the prototypic long pentraxin that was first identified in the early 1990s as a cytokine-inducible gene in endothelial cells and fibroblasts (1). Long pentraxins have an unrelated amino-terminal region coupled to a carboxy-terminal pentraxin-like domain. Experimental evidences indicated that PTX3, originally identified as an early induced protein in response to pro-inflammatory stimuli, is a soluble Pattern Recognition Molecule (PRM) involved in the humoral arm of the innate immune response (2, 3). However, in recent years several studies have shown that PTX3 is involved in other physiological and pathological processes such as tissue injury response, ectopic calcifications formation and in bone homeostasis (**Figure 1**). The human *PTX3* gene (MIM# 602492) is located on chromosome 3q25 and contains three exons and two introns. Mature PTX3 is characterized by a unique long N-terminal Domain (NTD - amino acids 18-179), and a pentraxin-like C-terminal domain (PTX - amino acids 179–381), homologous to the short pentraxins (4, 5). The molecule has a complex octameric structure composed of two covalently linked tetramers (6) and characterized by a peculiar asymmetric and elongated shape in which two domains are interconnected by a linear region (7). Hemostasis-related function of PTX3 in tissue repair and matrix formation has recently been described (8, 9). In several mouse models of tissue damage has been demonstrated that PTX3 promoted pericellular fibrinolysis in damaged site binding fibrin and plasminogen (Plg), thus ensuring appropriate tissue repair (10). Thus, in line with these studies, PTX3 plays an essential role in the orchestration of the tissue injury response. Furthermore, several studies demonstrate the important role played by PTX3 in ectopic calcification processes in extraosseous sites. Indeed, it has been seen to be involved in HA crystals formation and breast cancer cells differentiation into Breast Osteoblast Like Cells (BOLCs). Finally, recent investigations highlighted a possible role of PTX3 in osteoblast activity and bone matrix formation in both human and mouse tissues (11–14). Noteworthy, in these studies the impairment of PTX3 expression was correlated to the occurrence of osteoporosis and more in general with a significant decrease of osteoblast differentiation. Starting from these considerations, this narrative review aims to report the most recent data concerning the role of PTX3 in bone diseases.

## Role of PTX3 in Ectopic Calcifications

In recent years, several investigations highlighted the role of PTX3 in ectopic calcifications (**Table 1**). Specifically, Scimeca et al. demonstrated that the impairment of PTX3 expression inhibited the synthesis of HA (13). This result was also confirmed by *ex vivo* investigations that aimed to describe the molecular mechanisms involved in the ectopic calcifications’ formation. These elemental signs of human diseases are frequently detected in pathological tissues as consequence of cell homeostasis deregulation. The most common calcifications found in non-bone tissues are breast microcalcifications (21). Deposits of calcium salts in breast are associated to the presence of lesions, both benign and malignant. Thus, detection of microcalcifications in breast tissues by imaging analysis, such as echography, Magnetic Resonance Imaging (MRI) or most commonly by mammography, currently represents the main breast cancer screening investigation (21). After the pioneering studies of Morgan et al. (22–24), numerous investigations have been performed about the origin of breast microcalcifications. Our group proposed an innovative idea in which BOLCs developed into breast cancer mass are responsible of the production of HA crystals (15, 16, 25, 26). The progressive accumulation of these calcium salts induces the formation of calcified nodules detectable by mammography. One of the mechanisms that drive the differentiation of breast cancer cells into the BOLCs and also the formation of HA crystals is mediated by PTX3 (16). Specifically, we found that breast cancer tumors with a high BOLCs amount and high PTX3 expression were characterized by several HA microcalcifications (16). In addition, microcalcifications produced by BOLCs showed a histological aspect like an osteon. Noteworthy, we also found that the presence of PTX3-positive BOLCs significantly increases the risk of developing breast cancer metastasis to bone within 5 years from first histological diagnosis (27). Therefore, it is possible to hypothesize that PTX3 can induce osteoblastic differentiation and activity in both bone and extra-bone sites such as the breast. In this respect, we described the same mechanisms for the calcifications’ formation and bone metastasis in prostate cancer (28). A role of PTX3 was described also for the atheromatic lesions formation (29–31). However, to the best of our knowledge, no association has been identified between PTX3 expression and the formation of calcified nodules into the atheroma. Nevertheless, in our experience, the macrophages responsible for the atheromatic calcifications formation are frequently positive for PTX3 expression (32). Thus, it might be reasonable that osteoblastic differentiation process of atheromatic macrophages could in turn be driven by PTX3. It is important to remember that PTX3 is only one of the numerous possible mediators involved in the osteoblastic differentiation both in bone and ectopic sites, even though its activity seem to be fundamental for the mineralization process.

**Table 1:** Pentraxin 3 (PTX3) involvement in pathophysiological state of bone tissue’s metabolism.

Pathophysiological state	Species	System	Biological process	Reference
ECTOPIC CALCIFICATION	Human	BOLCs	Differentiation of breast cancer cells	(15)
	Human	BOLCs	Differentiation and microcalcifications formation	(16)
OSTEOLYTIC CONDITION OF INFLAMED BONE	Human	pOBs	Osteoclastogenesis induction by increasing RANKL production	(17)
FRACTURE HEALING	Mouse	Non-hematopoietic periosteal cells	Periosteal reaction induction during fracture repair	(18)
OSTEOPOROSIS	Human	OBs	Differentiation, proliferation and function	(13)
BONE METABOLISM	Human	Plasma	Inverse correlation between plasma level and BMD	(19)
FRACTURE REPAIR	Mouse	OBs, OCs	Matrix mineralization sequestering FGF2	(12)
BONE METABOLISM	Mouse	MC3T3-E1 cells	Signature genes (RUNX, ALP, OSC, OSX) overexpression	(20)

BOLCs, breast osteoblast like cells; pOBs, osteoblast’s precursor; OBs, osteoblasts; OCs, osteoclasts.

## PTX3, Osteoporosis, and Fracture Healing

Recent studies have demonstrated the importance of the role played by PTX3 in bone tissue metabolism (**Table 1**). Particularly, the involvement of this molecule in aging-related bone diseases, such as osteoporosis, and fracture healing process has been studied. Given its recently emerging role, attention has been focused on PTX3 involvement in bone homeostasis. Particularly, great interest has been shown for its involvement in the pathogenesis of chronic bone diseases such as osteoporosis and in the fracture healing process, both in mice and humans (13, 14, 17, 18, 20, 33). It has been shown that PTX3 is able to induce Receptor Activator of Nuclear factor Kappa-B Ligand (RANKL) expression by osteoblasts that promote the activation of osteoclasts in an *in vitro* culture system (17). Lee and colleagues speculated that PTX3 could perform a pathological function: its expression in bone can be elevated by TNF-α, which increases RANKL expression by osteoblast’s precursors, that, in turn, induces an excessive formation of osteoclasts. These results suggest that PTX3 can act as an inflammatory mediator that contributes to the deterioration of the osteolytic conditions of the inflamed bone. On the other hand, it has been proposed that PTX3 is involved in bone formation rather than resorption, as demonstrated by Kelava et al. in 2014, who studying the relationship between PTX3 expression and bone quality in mice deficient for the *PTX3* gene, demonstrated that PTX3 null mice had lower bone mass than wild-type mice (18). To establish the effective involvement of PTX3 in bone pathophysiology, its expression and function in human osteoblasts of osteoporotic, osteoarthritic patients and young subjects not affected by bone pathologies were studied (14). Immunohistochemical analysis performed on bone head biopsies showed a close association between bone health and the number of osteoblasts expressing PTX3. It was observed that the percentage of PTX3 positive osteoblasts was significantly lower in osteoporotic patients than in young and osteoarthritic patients of the same age (13). Furthermore, the treatment with an anti-PTX3 antibody of human osteoblast primary cultures derived from young patients has greatly influenced the behavior of osteoblasts: they lost the morphological and molecular characteristics typical of mature osteoblasts, acquiring fibroblast-like shape and strongly decreasing RANKL and RUNX2 expression. Finally, PTX3 inhibition negatively influenced osteoblasts proliferation and their ability to form cell groups and HA microcrystals. Overall, it has been hypothesized that bone regeneration requires PTX3 both for the induction of the fracture healing process and for the recruitment of differentiated osteoblasts (14). Some researchers are testing the possibility to use PTX3 plasma levels as a biomarker for the bone quality and to stratify the population based on the risk to develop an osteoporotic condition. In this context, a paradox effect has been described by Lee and colleagues (19). In particular, authors evaluated PTX3 plasma levels on a sub-cohort of 1440 subjects (757 men and 683 women) founding that PTX3 levels were inversely associated with Bone Mineral Density (BMD) of the lumbar spine and femoral neck in men but not in women (19). This data could be explained as a systemic reaction of the body to offset the impairment of PTX3 in specific tissue as the bone. In agreement with these evidences, Grčević and colleagues performed an *in vivo* study in which PTX3 KO mice (*Ptx3-/-*) was used to test the role of PTX3 in bone turnover and repair (12). By using the closed transversal tibial fracture model, authors found that *Ptx3-/-* female mice formed significantly less mineralized callus during the anabolic phase following fracture injury compared to wild-type mice. These results indicate that PTX3 plays an important role in bone homeostasis and in proper matrix mineralization during fracture repair, a reflection of this molecule function in tissue homeostasis and repair (12). Moreover, PTX3 can interact with Fibroblast Growth Factor 2 (FGF2), which is highly expressed in the bone matrix, exerting a negative effect on osteoblastogenesis and bone remodeling. It has been demonstrated *in vitro* that PTX3 produced by osteoblasts is able to bind FGF2 and inhibit the negative effects exerted on the differentiation of bone marrow stromal cells into osteoblasts, acting as a protective factor for bone (33). Finally, a study performed by Liu et al. investigated the role played by PTX3 in promoting osteoblastic differentiation in MC3T3-E1 cells, confirming the essential role of PTX3 in the bone metabolism (20). Authors showed that PTX3 is abundantly expressed in MC3T3-E1 cells and that its expression is inducible by the introduction of osteogenic induction medium. In addition, the overexpression of PTX3 was able to significantly increase the expression of some of the most important genes related to bone metabolism such as Runt-related Transcription Factor 2 (RUNX2), ALP, Osteocalcin (OCN) and Osterix (OSX), suggesting that the PTX3 overexpression promotes osteoblastic differentiation and activity (20). As concern the possible molecular pathways involved in the bone metabolism regulation by PTX3, it is demonstrated that the effects of PTX3 on osteoblasts are mediated by its induction of the PI3K/Akt signaling pathway. From mechanistic point of view, Liu et al. displayed that the action of PTX3 requires the activation of PI3K and Akt, and deactivation of PI3K by its inhibitor LY294002 weakens the PTX3-mediated induction of osteoblast signature genes, ALP and matrix mineralization (20). These data provide the rationale to develop new strategies for bone metabolism regulation in human subjects. Indeed, the possibility to modulate the molecular signaling involved in the PTX3-related osteoblast differentiation could represents an opportunity for future innovative therapies for osteoporotic patients.

## PTX3 Genetic Variability and Aging-Related Bone Diseases

In recent years, increasing research has focused on the association between the Single-Nucleotide Polymorphisms (SNPs) of *PTX3* gene and human diseases. However, there are still few reports about the effect of PTX3 genetic variability and aging-related bone diseases. Previous studies have described that rs2305619 and rs1840680 SNPs have functional significance on the relative PTX3 protein amount. Results indicated that the A allele of both SNPs is associated with higher levels of circulating PTX3 (34, 35). The mechanism by which these SNPs affect PTX3 plasma levels has still to be clarified but possibly rs2305619 and rs1840680 are in linkage disequilibrium with a genetic variant located in a regulatory region of the gene. Variable levels of PTX3, depending on different genetic variants, may therefore have a direct role in the pathogenesis of inflammation and ossification. On this basis, Zhang and Ding (36) analysed the effects of three PTX3 polymorphisms in genetic susceptibility to Ankylosing Spondylitis (AS). AS is a complex inflammatory disease characterized by a chronic long-term inflammation of the joints of the spine over time. As an autoimmune disease, AS develops through complex interactions between the individual genetic background and environmental factors (37). Genotyping of rs23056219, rs3816527, and rs3845978 SNPs in *PTX3* gene has been conducted by allelic discrimination assay in 101 AS patients and 93 controls. Results showed that the C allele and the CC genotype of rs3816527 have a positive effect on AS occurrence. Similarly, in rs3845978, individuals carrying the T allele and the CT genotype developed AS earlier. The three SNPs belong to a haplotype block and their combined analysis revealed that G-C-T and A-C-C haplotypes may increase the genetic susceptibility to AS (36). Another genetic aspect linked to the increased risk to develop bone disease is the so called “inflamm-aging”, the age-related elevations in proinflammatory cytokines associated with shorter immune cell telomere lengths. Telomeres maintain the “youthful” status of immune cells, and shortened telomere lengths are considered a main biological hallmark of cellular aging (38) leading to an irreversible state of replication-induced cellular senescence (39). Although leukocyte telomere lengths shorten as a natural consequence of aging, the continuous exposure to increased proinflammatory agents may accelerate this process (40). It has been demonstrated that increased PTX3 production is required to prevent overactivation of the inflammatory signalling pathway, and elevated concentrations of circulating PTX3 are considered an indicator of appropriate immune function in healthy young adults (41). This observation has been further confirmed in the study by Pavanello et al (42). who recently demonstrated that the upregulation of PTX3 plasma levels is associated with longer telomere lengths in healthy middle aged adults (41). Last but not least, epigenetic mechanisms have been also implicated in the regulation of human PTX3 expression, since methylation of 5’ UTR regions (enhancers and promoter) has been deemed responsible of PTX3 gene silencing in several disease conditions including cancer, inflammation and atherosclerosis (43, 44). Chronic inflammation induces a novel epigenetic program that is conserved in intestinal adenomas and in colorectal cancer (45). It is therefore possible to speculate that the persistent inflammatory stimuli from an aged immune system may be also involved in the progression of age-related bone diseases through either genetic (telomere shortening) and epigenetic (DNA hypermethylation) mechanisms acting on the *PTX3* gene.

## Discussion

In this systematic review, we discussed and summarised the effects of PTX3 on various biological mechanisms, focusing our attention on the role played by this protein in the physiology and pathophysiology of mineralization process, in bone formation and ectopic calcifications, as well as its involvement in the onset of age-related bone diseases. In fact, PTX3 seems to be involved in tissue remodelling and repair processes, both under physiological and pathological conditions (1, 33). In this regard, our group demonstrated for the first time the ability of PTX3 to induce an increase of both cell proliferation and HA microcrystal formation in osteoblasts culture characterized by an impairment of PTX3 expression. These results allowed us to speculate the existence of a close correlation between PTX3 expression by osteoblasts and bone quality, suggesting a central role for PTX3 in proliferation, differentiation, and function. The ability of PTX3 to participate in HA microcrystals’ formation has also been confirmed by *ex vivo* studies to describe the molecular mechanisms involved in ectopic calcifications’ formation, which are often detected in pathological tissues, resulting in an altered cellular homeostasis. PTX3 is known to mediate the differentiation of breast cancer cells into BOLCs cells and the formation of HA crystals (26). Based on these evidences, PTX3 can be considered a new possible marker for poor differentiated breast cancer (46). Furthermore, since a significantly greater difference in PTX3 expression between malignant and benign lesions in the presence of microcalcifications was found, it can be assumed that PTX3 plays an essential role in the formation of ectopic calcifications in breast. Besides being considered one of the many possible mediators involved in osteoblastic differentiation both in bone and ectopic sites, several studies agree with the definition of PTX3 also as a molecule significantly involved in the pathogenesis of age-related bone diseases, such as osteoporosis, and in the fracture healing process. In this regard, to establish the effective involvement of PTX3 in bone pathophysiology, Scimeca and colleagues studied the expression and function of this protein in osteoblasts from osteoporotic and osteoarthritic patients and young subjects not affected by bone diseases (13). It is important to highlight the role of PTX3 in bone regeneration, since its presence would appear to be crucial both for inducing the fracture healing process and recruitment of differentiated osteoblasts. In recent years, it has been assumed that the onset and progression of age-related bone diseases are also influenced by genetic and epigenetic mechanisms acting on the *PTX3* gene. Indeed, it is possible to speculate that genetic variability, together with epigenetic modifications, in PTX3 regulatory regions could lead to an alteration of PTX3 levels and may be directly involved in the pathogenesis of inflammation and ossification. Based on these scientific evidences, we can certainly conclude that PTX3 may represent a new regulator of bone metabolism. Given its evident involvement in the physiological and pathophysiological processes that occur in bone tissue, PTX3 could represent a new potential diagnostic marker and therapeutic target for the treatment of age-related bone diseases, representing an important goal in the era of personalized medicine. Considering the multiple roles played by PTX3 in both physiological and pathological contexts, we believe it is necessary to address future studies towards a better and deeper understanding of the underlying mechanisms, in order to make possible the development of targeted therapeutic approaches. Finally, we believe that the use of PTX3 as a marker can contribute significantly to a reduction in costs associated with diagnostic procedures and treatment of bone metabolism disorders.

## Author Contributions

CG, VV, IC, AB, MC, MG, and MS wrote the initial draft of the manuscript. UT, EG, MS, and AS contributed to the editing and revising of this work. All authors contributed to the article and approved the submitted version.

## Conflict of Interest

The authors declare that the research was conducted in the absence of any commercial or financial relationships that could be construed as a potential conflict of interest.
